# The evaluation of ADME and pharmacokinetic properties of decoquinate derivatives for the treatment of malaria

**DOI:** 10.3389/fphar.2022.957690

**Published:** 2022-08-19

**Authors:** Daniel J. Watson, Lizahn Laing, Richard M. Beteck, Liezl Gibhard, Richard K. Haynes, Lubbe Wiesner

**Affiliations:** ^1^ Division of Clinical Pharmacology, Department of Medicine, University of Cape Town, Cape Town, South Africa; ^2^ Centre of Excellence for Pharmaceutical Sciences, School of Health Sciences, North-West University, Potchefstroom, South Africa; ^3^ Department of Chemistry, University of Cape Town, Cape Town, South Africa

**Keywords:** quinolones, decoquinate, antimalarial activities, pharmacokinetics, ADME

## Abstract

The emergence of *Plasmodium falciparum* (*Pf*) parasite strains tolerant of the artemisinin component and resistant to the other drug component in artemisinin combination therapies (ACTs) used for treatment now markedly complicates malaria control. Thus, development of new combination therapies are urgently required. For the non-artemisinin component, the quinolone ester decoquinate (DQ) that possesses potent activities against blood stage *Pf* and acts on a distinct target, namely the *Pf* cytochrome *bc*_1_ complex, was first considered. However, DQ has poor drug properties including high lipophilicity and exceedingly poor aqueous solubility (0.06 μg/ml), rendering it difficult to administer. Thus, DQ was chemically modified to provide the secondary amide derivative RMB005 and the quinoline *O*-carbamate derivatives RMB059 and RMB060. The last possesses sub-nanomolar activities against multidrug resistant blood stages of *Pf*, and *P. berghei* sporozoite liver stages. Here we present the results of ADME analyses *in vitro* and pharmacokinetic analyses using C57BL/6 mice. The amide RMB005 had a maximum mean whole blood concentration of 0.49 ± 0.02 µM following oral administration; however, the area under the curve (AUC), elimination half-life (t_1/2_) and bioavailability (BA) were not significantly better than those of DQ. Surprisingly, the quinoline *O*-carbamates which can be recrystallized without decomposition were rapidly converted into DQ in human plasma and blood samples. The maximum concentrations of DQ reached after oral administration of RMB059 and RMB060 were 0.23 ± 0.05 and 0.11 ± 0.01 µM, the DQ elimination half-lives were 4.79 ± 1.66 and 4.66 ± 1.16 h, and the DQ clearance were 19.40 ± 3.14 and 21.50 ± 3.38 respectively. Under these assay conditions, the BA of DQ could not be calculated Overall although RMB059 and -060 are labile in physiological medium with respect to the DQ parent, the potential to apply these as prodrugs is apparent from the current data coupled with their ease of preparation.

## 1 Introduction

Whilst incidence of malaria has steadily declined since 2000, 241 million new cases of malaria were reported in 2020 (World Health Organization 2021). This persistently high infection rate highlights the need to improve control and treatment measures. Unfortunately, effective treatment is now hampered by increasing tolerance of *Plasmodium falciparum* (*Pf*) to the artemisinin component, and formal resistance to the second drug comprising artemisinin-based combination therapies (ACTs) (Ippolito et al., 2021). Efforts are underway to find new drugs for combination therapies in order to counter drug resistance, and additionally, the use of triple artemisinin combination therapies (TACTs) have been proposed (van der Pluijm et al., 2020). We have described elsewhere our own efforts to develop TACTs based on discrete consideration of mechanism of action of the components (Coertzen et al., 2018; Wong et al., 2020). We focus on newer amino-artemisinin derivatives that are pan-reactive against asexual and transmissible blood stage parasites, a redox active second drug such as methylene blue (Akoachere et al., 2005), phenoxazine (Schleiferböck et al., 2013) or naphthoquinone (Ehrhardt et al., 2016) which displays synergism with the artemisinins, and a longer half-life third drug based on a distinct target that should help to expunge artemisinin-tolerant parasites.

**FIGURE 1:**
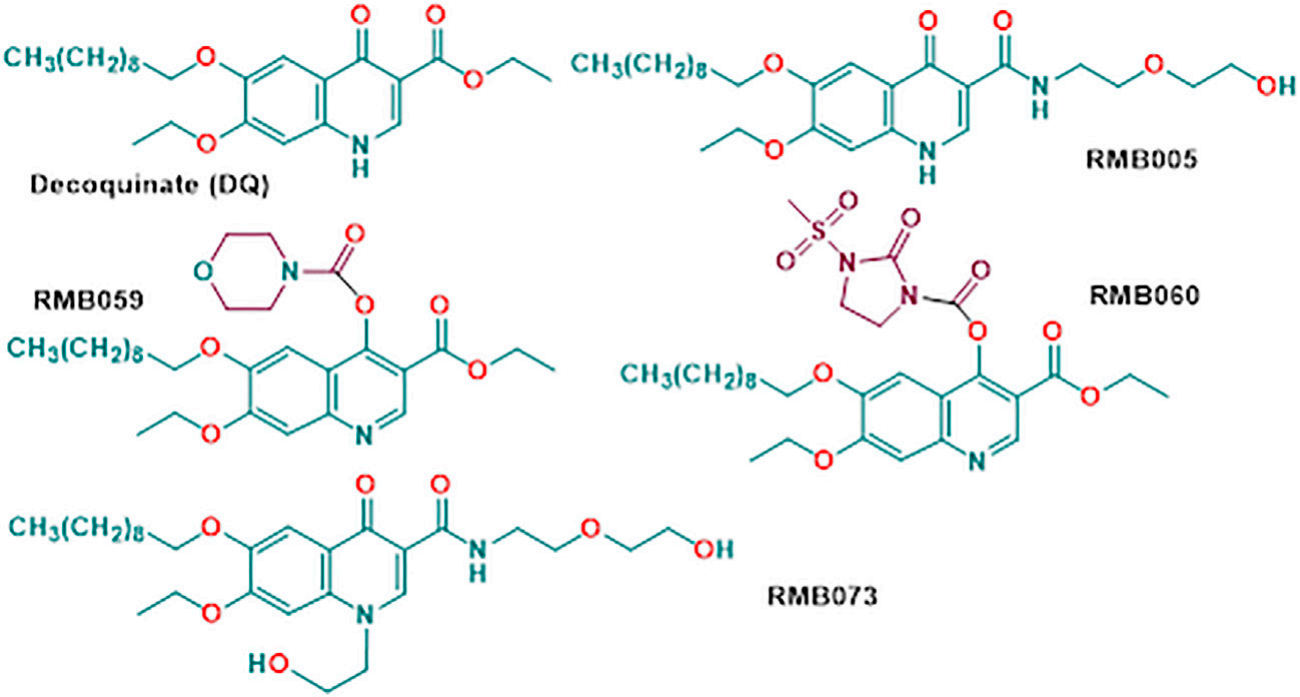
Structures of decoquinate and the antimalaria-active quinolone derivatives RMB005, RMB059 and RMB060. Compound RMB073 is a stable TB-active DQ derivative (Beteck et al., 2018) whose DMPK parameters have been determined (Tanner et al., 2019) and which is used here as an internal standard during determination of whole-blood concentrations of the malaria-active compounds.

For the last, we considered quinolones based on decoquinate (DQ) (Beteck et al., 2018). DQ was first described in 1968 (Anadón and Martínez-Larrañaga 2014) and is widely used as a coccidiostat that is added to feed in animal husbandry practice (Taylor and Bartram 2012). Like other quinolones, it displays antimalarial activity (Ryley and Peters 1970) wherein the mechanism of action involves blockade of the quinol reductase site of the parasite mitochondrial cytochrome *bc*_*1*_ complex (Cruz et al., 2012). DQ possesses nanomolar *in vitro* activities against chloroquine-sensitive NF54 asexual parasites (26.6 nM) (Beteck et al., 2016) and is also active against liver and sexual gametocyte stages of *Pf*. While DQ shows limited cross-resistance to atovaquone resistant parasites and prophylactic efficacy as a single oral dose (Nam et al., 2011; Wang et al., 2014), its notably low aqueous solubility (0.06 µg/ml; 0.01 µg/ml in buffered water within a pH range of 4–9) (EFSA 2003) low bioavailability and high lipophilicity renders DQ difficult to administer, and considerable effort has been expended in developing either special release formulations suitable for oral administration (Wang et al., 2013; Wang et al., 2014; Zeng et al., 2022) or appending highly polar groups to generate DQ prodrugs with enhanced water solubility (Pogany et al., 2012). A notable example of use of the prodrug concept to enhance bioavailability of the quinolone ELQ-300 is provided by its conversion into the quinoline carbonate ELQ-337 which upon oral administration in a mouse model provides the parent drug ELQ without detectable persistence of the intact prodrug in murine plasma; however, greatly enhanced levels of ELQ-300 are observed through administration of ELQ-337 (Miley et al., 2015). In our case, DQ was converted by simple amide-ester exchange into the secondary amide RMB005, or by direct treatment with the corresponding carbamoyl chlorides into the quinoline *O*-carbamate derivatives RMB059 and RMB060 (Beteck et al., 2018) (Figure 1). The amide RMB005 displays activities similar to those of DQ against *Pf* NF54 (IC_50_ 40.4; DQ 26.6 nm) and multidrug resistant *Pf* K1 (IC_50_ 64.8, DQ 64.9 nm) (Beteck et al., 2018). Both RMB059 and RMB060 are more active in than DQ displaying IC_50_ activities of 6.8 and 1.4 nm against *Pf* NF54, and 14.4 and 1.3 nm respectively against *Pf* K1. Against multidrug resistant *Pf* W2, activities are 5.7 and 1.1 nm. Additionally, RMB060 is potently active *in vitro* against the apicomplexan parasites *Toxoplasma gondii* (*Tg*) and *Neospora caninum* with IC_50_ values of 1.1 and 0.6 nm respectively (Beteck et al., 2018). Thus, these quinoline carbamates have superior activity profiles to DQ, and are not appreciably cytotoxic, displaying selectivity indices of >528 for *Pf* vs. CHO cells and ∼4,000 for *Tg* vs. host HFF cells (Beteck et al., 2018). Structurally distinct derivatives of DQ bearing *N*-alkyl groups on the quinolone nitrogen and amide replacing the ethyl ester are potently active against *Mycobacterium tuberculosis*, the causative agent of tuberculosis, although these latter derivatives are not appreciably active against malaria (Beteck et al., 2018). One such compound RMB073 is shown in Figure 1.

We have now submitted RMB005, −059 and −060 to ADME assays including determination of aqueous solubilities, permeability, lipophilicity, plasma and metabolic stabilities, protein binding, and have evaluated pharmacokinetic (PK) properties using a murine model. The methods were used previously for determining drug metabolism and PK profiles of the TB-active decoquinate derivatives including the *N*-alkyl quinolone amide RMB073 (Figure 1) (Beteck et al., 2018; Tanner et al., 2019).

## 2 Materials and methods

### 2.1 Ethics statement

All animal studies and procedures were conducted with prior approval of the Ethics Committee of University of Cape Town (approval number 013/028) in accordance with the South African National Standards (SANS 10386:2008) for the Care and Use of Animals for Scientific Purposes, (*South African National Standard: The Care and Use of Animals for Scientific Purposes* South African National, 2008) and guidelines from the Department of Health (Department of Health 2015).

### 2.2 Chemicals and reagents

Compounds RMB005, −059, −060, and −073 were prepared and purified (>96%) as previously described, with purity being determined by high-performance liquid chromatography (HPLC) (Beteck et al., 2018). Acetonitrile, potassium dihydrogen phosphate and dipotassium hydrogen phosphate were purchased form Merck (Darmstadt, Germany). Analytical grade dimethyl sulfoxide (DMSO), formic acid, carbamazepine, hydrocortisone, propranolol hydrochloride, verapamil and vinpocetin were obtained from Sigma-Aldrich (St Louis, MO, United States). Water was purified by Millipore Elix 10 reverse osmosis and a Milli-Q (Millipore, United States). Human plasma was obtained from the Western Province blood transfusion services (Cape Town, South Africa) and liver microsomes were obtained from Xenotech (Kansas City, KS, United States). All other reagents were of analytical grade.

### 2.3 ADME assays

#### 2.3.1 Kinetic solubility

Stock concentrations of test samples (10 mM) were prepared in DMSO and diluted to a final concentration of 200 mM in phosphate buffer saline (PBS) solutions at pH 2, 6.5, and 7.4 in flat-bottomed 96-well plates. A three-point calibration curve of each control and test compound was prepared from stock solutions in DMSO. Plates were agitated on an orbital shaker at room temperature for 2 h at 200 rpm and subsequently analysed using a reverse phase Gemini NX-C18 column (5 μM, 2.1 mm × 50 mm) with an Agilent 1,200 Rapid Resolution HPLC and diode array detection.

#### 2.3.2 Lipophilicity

Stock solutions (10 μM) of compounds were diluted to 100 μM in 96-well deep-well plate containing 1-octanol and phosphate buffer at pH 7.4. The plate was agitated on an orbital shaker at 750 rpm for 2 h. The organic and buffer layers of each well were transferred separately to a new analysis plate. The samples were analysed with the HPLC-diode array detection as described for kinetic solubility. Samples from both layers were analysed to determine LogD_7.4_ values.

#### 2.3.3 Passive permeability

A parallel artificial membrane permeation assay (PAMPA) was used to assess the passive permeability of compounds with a 96-well MultiScreen filter plate (0.4 μM pore size, Millipore). The filter plate was precoated with 5% hexadecane in hexane. Stock solutions of compounds were diluted to 1 mM in a donor buffer (pH 6.5). Lucifer yellow was added to the apical wells of the precoated MultiScreen plate containing compound donor solution. The donor buffer solutions were spiked with each test compound (1 mM). The acceptor-buffer solution was prepared by adding 10 μL DMSO to 990 μL acceptor buffer (pH 7.4) and 250 μL were added to the basolateral (acceptor) wells. The donor plate was carefully inserted into the acceptor plate and incubated at room temperature for 4 h. After the incubation period, 50 μL of samples from the acceptor plate were transferred to a round bottom 96-well plate, together with 30 μL of donor buffer for matrix matching. Theoretical equilibrium samples for each test compound were prepared by adding 150 μL of donor solutions to 250 μL acceptor solutions and left at room temperature. These theoretical equilibrium samples represent complete transfer of the compound into the donor well. For the theoretical equilibrium samples, 80 μL were added to the analysis plate. Lastly, 160 μL of the internal standard carbamazepine (0.1 μM) was added to all acceptor and equilibrium samples. A portion of the sample containing Lucifer yellow was analyzed on a BioRad iMark^TM^ Microplate Absorbance Reader (BioRad, Hercules, CA, United States; excitation 490 nm, emission 510–570 nm) to determine that *P*_app_ was within the acceptable range (<50 nm/s). Samples were analysed using reverse phase Gemini NX-C18 column (5 μM, 2.1 mm × 50 mm) with a Shimadzu HPLC coupled with an AB Sciex 3200 Q TRAP MS). The P_app_ for each compound was determined using.

$$C=\frac{V_{D\;}\times \;V_{A}}{\left(V_{D}+V_{A}\right)\;\times A\times t\;}$$

where V_A_ = volume of donor compartment (0.15 cm^3^), V_D_ = volume of acceptor compartment (0.25 cm^3^), A = accessible filter area (0.24 cm^2^) and t = incubation time (14,400 s).

Lucifer yellow permeability value (P_app_)

$$P_{app}=C\times -\ln\left(1-\frac{\left[Acceptor\;well_{absorbance}\right]}{\left[Donor\;well_{absorbance}\right]}\right)$$

Compound permeability value (P_app_)

$$P_{app}=C\times -\ln\left(1-\frac{\left[Acceptor\;well_{peak\;area}\right]}{\left[Donor\;well_{peak\;area}\right]}\right)$$

#### 2.3.4 Plasma stability

Stock solutions of compounds in DMSO were used to spike pooled human plasma. Samples were transferred in duplicate to 6 different wells of a 96-deep well plate. An aliquot of each sample was immediately quenched with ice cold acetonitrile containing the internal standard carbamazepine (0.1 μM), after which the plate was incubated in a water bath (37°C for 1 h). Aliquots of samples were removed at 5, 15, 40, 60, and 180 min and quenched with ice cold acetonitrile. After samples of the final time point were precipitated, the supernatant was transferred to a round bottom 96-well plate for analysis by LC-MS/MS using a reverse phase Gemini NX-C18 column (5 μM, 2.1 mm × 50 mm) with a Shimadzu HPLC coupled with an AB Sciex 3200 Q TRAP MS). The peak area ratios were used to calculate the amount of parent compound remaining and t_1/2_ in plasma, using the following equations.

% Parent remaining

$$\%\;Parent=\;\frac{Normalised\;peak\;area\;of\;sample\;at\;timepoint}{Normalised\;peak\;area\;of\;sample\;at\;t=0}\;\text{x}\;100$$

Predicted t_1/2_

t1/2= −0.693/λ

Where λ is the slope of the Ln % parent remaining vs. time curve.

#### 2.3.5 Plasma protein binding and non-specific liver microsomal binding

Plasma protein binding was determined in pooled human plasma using an ultracentrifugation method. Stock solutions of test compounds were diluted in phosphate buffer and spiked into plasma in deep well 96-well plates. An aliquot of each compound was immediately precipitated with ice cold acetonitrile containing the internal standard carbamazepine (0.1 μM). These served as total concentration samples. After incubation in a water bath (37°C for 1 h), samples were transferred to ultracentrifuge tubes in duplicate and centrifuged for 4 h at 37°C and 30,000 g (Optima L-80XP, Beckman). Following centrifugation, the supernatant was transferred to the plate containing the total concentration samples and precipitated with ice cold acetonitrile containing the internal standard. Samples were analysed by LC-MS/MS (Shimadzu HPLC coupled with an AB Sciex 3200 Q TRAP MS). Non-specific binding in mouse liver microsomes (MLM) and human liver microsomes (HLM) were also determined with the ultracentrifugation method. Stock solutions of compounds (10 mM) were added in duplicate to microsome solution (0.5 mg/mL) in phosphate buffer (pH 7.4) to obtain a final drug concentration of 1 μM. The same method as for plasma protein binding was utilized. Analyte concentrations of all compounds were determined via LC-MS/MS (reverse phase Gemini NX-C18 column and Shimadzu HPLC coupled with an AB Sciex 3200 Q TRAP MS).

#### 2.3.6 Metabolic stability

A 5-point metabolic stability assay in human and mouse liver microsomes were performed in duplicate in 96-well plates. Stock solution of RMB005, −059 and 060 prepared in DMSO were individually incubated at 37°C in mouse and pooled human liver microsomes (0.4 mg/mL). An aliquot was removed at 0 min and quenched with ice cold acetonitrile containing the internal standard carbamazepine (0.1 μM). The cofactor NADPH was added, and the reactions were subsequently stopped at time points 5, 10, 30, and 60 min by removal of an aliquot and quenching with ice cold acetonitrile containing the internal standard. The supernatant of all samples were analysed via LC-MS/MS analysis (reverse phase Gemini NX-C18 column and Shimadzu HPLC and AB Sciex 3200 Q TRAP MS). Calculation of *in vitro* t_1/2_, intrinsic clearance rate (CL_int_) and predicted *in vivo* clearance (CL_H_) was carried out using the following equations (Obach 1999).

$${\mathrm{CL}}_{\mathrm{int}}Cl_{int}=\;\frac{0.693}{t_{1/2}\left(min\right)}\;\times \frac{Volume\;of\;incubation\;\left(\mathrm{\mu L}\right)}{microsomal\;protein\;\left(\mathrm{\mu g}\right)}\;$$

Where t_1/2_ is calculated in minutes.

Predicted *in vivo* CL_H_

$$CL_{H}=\frac{Q\;\times \;f_{u\left(plasma\right)}\times \frac{CL_{int}}{f_{u\left(mic\right)}}}{Q+\;f_{u\left(plasma\right)}\times \;\frac{CL_{int}}{f_{u\left(mic\right)}}}$$

Where Q is the hepatic blood flow of mouse (90 mL/min/kg) or human (20.7 mL/min/kg). F_u(plasma)_ is the unbound drug fraction in the plasma and f_u(mic)_ is the unbound fraction in the microsomes.

The equation below was used in the case of RMB059 and -060, where protein-binding data were not available.

$$CL_{H}=\frac{Q\;\times \;CL_{int}}{Q+\;CL_{int}}$$

### 2.4 *In vivo* pharmacokinetic studies

#### 2.4.1 Animals

Healthy C57BL/6 mice each weighing approximately 25 g were maintained at the University of Cape Town animal facility. Mice were housed in 27 × 21 × 28 cm cages (*n* = 3) under controlled environmental conditions at 22 ± 2°C, humidity of 55 ± 15% and a 12-hour light/dark cycle. Food and water were available *ad libitum.* Mice were acclimatised to the experimental environment for 4–5 days prior to initiating the experiments.

#### 2.4.2 Oral drug administration

RMB005, −059 and −060 were prepared in an aqueous solution of HPMC containing 0.2% Tween 80. The weighed compound was sonicated and vortexed to obtain a homogenous suspension. Oral dosing of 20 mg/kg was achieved via oral gavage. The total volume per administration was 200 μL. Blood samples were collected post-dose in lithium heparin microvials *via* tail bleeding at predetermined intervals at 0.5, 1, 3, 5, 8, 10, and 24 h. Samples were gently vortexed and stored at −80°C until analysis.

#### 2.4.3 Intravenous administration

RMB005, −059 and −060 were prepared in an organic vehicle consisting of 10% *N*,*N*-dimethylacetamide, 30% polyethylene glycol 400 (PEG), 50% polypropylene glycol (PPG) and 10% ethanol (1:3:5:1, v/v) for intravenous 4) dosing at 10 mg/kg. The total volume of administration was 80 μL. Blood samples were collected post-dose in lithium heparin microvials via tail bleeding at predetermined intervals at 0.16, 0.5, 1, 3, 5, 8, 10, and 24 h. Samples were gently vortexed and stored at −80°C until analysis.

#### 2.4.4 Sample processing and analysis

A quantitative LC-MS/MS assay was used to determine whole-blood concentrations of RMB005, −059 and −060. Whole blood samples (20 μL) were treated with 100 μL ice cold acetonitrile containing the compound RMB073 used previously for determination of whole blood concentrations in analysis of TB-active compounds, as internal standard (200 ng/ml) (Tanner et al., 2019). The mixture was vortexed vigorously for 1 min, and 5 µL of the supernatant were injected onto the analytical column following centrifugation (5,590 g for 5 min). Gradient elution was carried out using a Gemini NX-C18 analytical column (5 μm, 2.1 mm × 50 mm). The aqueous mobile phase consisted of 5 mm ammonium acetate with 0.1% acetic acid in deionised water, while the organic phase consisted of 0.1% acetic acid in acetonitrile. A Shimadzu HPLC and reverse phase Gemini NX-C18 column coupled to an AB Sciex 3200 Q TRAP MS was operated at unit resolution in the multiple reaction monitoring (MRM) mode. The precursor ions, product ions and mass spectrometer conditions are summarised in Table 1. The transition ions for DQ were also included given that conversion to the parent compound was shown to take place, as indicated below. The calibration standards ranged from 2 ng/mL to 3,000 ng/mL for RMB005, and 2 ng/mL to 4,000 ng/mL for RMB059, −060 and DQ.

**TABLE 1:** Multiple reaction monitoring transitions and final mass spectrometer conditions.

Analyte	Transition, m/z	Declustering potential, V	Entrance Potential,V	Collision Energy,V	Cell Exit Potential,V
RMB005	475 → 334	−110	−10	−34	−4
	475 → 277	−110	−10	−58	−4
RMB059	531 → 114	111	9.5	41	2
	531 → 71.2	111	9.5	69	2
RMB060	608 → 204	121	12	85	4
	608 → 372	121	12	36	4
DQ	418 → 372	56	11	29	6
	418 → 203	56	11	57	4

### 2.5 Data analysis

Drug concentration versus time plots for each compound were used to determine maximal drug concentration *C*_max_, time *T*_max_ to reach *C*_max_, elimination half-life *t*_1/2_ and the area under the concentration-time curve from time zero to infinity, AUC_0-inf_. From these values the following PK parameters—clearance CL, volume of distribution (Vd) and oral bioavailability (BA) were determined using the non-compartmental analysis Microsoft Excel Add-In PKSolver (Zhang et al., 2010).

## 3 Results

### 3.1 ADME properties

Predicted kinetic solubilities, lipophilicity and permeability properties of RMB005, -059 and -060 are summarised in Table 2. Whilst RMB005 and RMB059 have a solubility of <5 μM at all pH levels, the relative instability of the quinoline *O*-carbamate RMB060 in solution precluded reliable evaluation of solubility. In contrast, the *N*-alkylquinolone RMB073 is stable, and like other *N-*alkylquinolones derived from DQ, has a predicted kinetic solubility of >150 µM at pH 7.4 (Tanner et al., 2019). Thus, as exemplifed by the low solubility of RMB005, attachment of an alkyl group to N-4 of the quinolone greatly enhances solubility. The distribution coefficients of the compounds in 1-octanol and buffer at pH 7.4 (LogD_7.4_) used as a measure of lipophilicity are given in Table 2.

**TABLE 2:** Predicted solubilities, lipophilicities and permeabilities *in vitro*.

Compound	Kinetic solubility (μM)			Lipophilicity (LogD_7.4_)	Permeability (LogP_app_)	Plasma half-life (min)
	pH 2	pH 6.5	pH 7.4			
RMB005	<5	<5	<5	3.2	−6.3	>150
RMB059	<5	<5	<5	2.9	−5.8	66
RMB060	nd	nd	nd	2.9	−6.3	<8

nd, not determined.

Whilst the kinetic solubility of DQ was not determined, it has been reported that solubility of DQ is 0.01 µg/mL or 0.024 µM in buffered aqueous solutions within the pH range of 4–9 (EFSA 2003). In addition, the octanol/water partition coefficient of DQ at 25°C (Log K_ow_ or LogP) is ≥5.7 at pH 5, 7, or 9 (Bampidis et al., 2019). Thus, in so far as a direct comparison can be made, lipophilicity parameters for DQ are not significantly different to those of the derivatives. Likewise, aqueous solubilities in so far as these can be determined for RMB005 and RMB059 are not significantly different to that of DQ.

**FIGURE 2:**
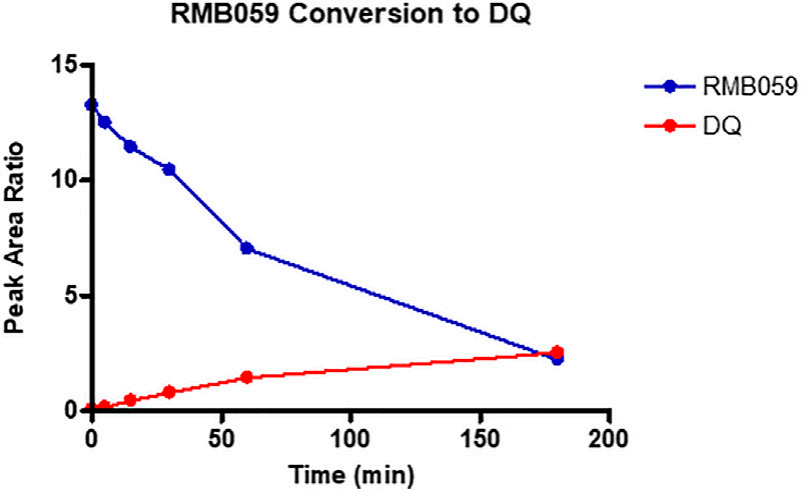
Conversion of RMB059 into DQ during 3-hour incubation in human plasma.

**FIGURE 3:**
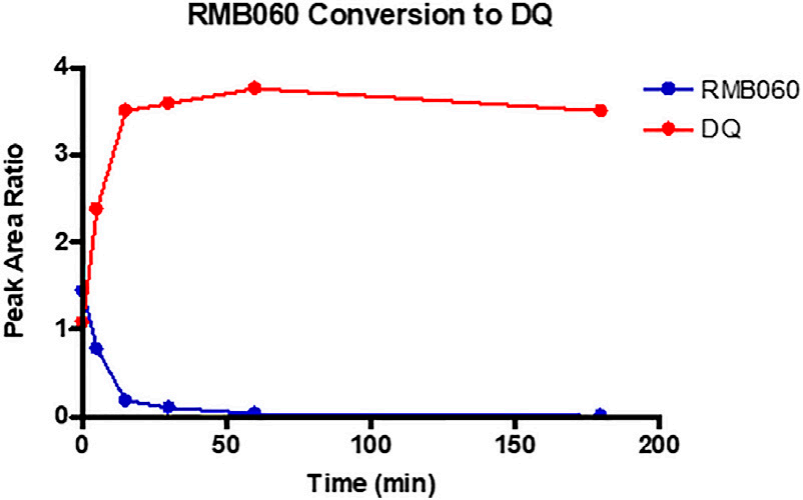
Conversion of RMB060 into DQ during 3-hour incubation in human plasma.

For assessing half lives in plasma, as decomposition of the quinoline *O*-carbamates to DQ takes place in plasma, the transition ions of DQ were included in the LC-MS/MS detection methods. Thereby the generation of DQ via decomposition of RMB-059 and −060 was able to be followed. The results are depicted in Figures 2, 3. The instability of RMB060 was unexpected—it can be purified by chromatography using protic solvents and recrystallized without decomposition (Beteck et al., 2018). However, as noted above, the structurally related quinoline *O*-carbonate prodrug ELQ-337 undergoes rapid hydrolysis in murine plasma to the parent quinolone ELQ-300 in much the same way as RMB059 and -060 form DQ (Miley et al., 2015). In contrast, the amide RMB-005 is relatively stable in plasma, as are *N*-alkylquinolones such as RMB-073 and others, with plasma half-lives greater than 6 h (Tanner et al., 2019).

The metabolic stability of RMB005, −059 and −060 were evaluated by calculating the intrinsic clearance with human and mouse microsomes. The t_1/2_ so derived was then used to determine CL_int_ and CL_H_; these values are listed in Table 3. RMB005 was highly bound to microsomes (99% for both species, respectively) and moderately bound to plasma proteins (92%). However, protein binding of each of RMB059 and RMB060 could not be carried out, due to their susceptibility to degradation in plasma.

**TABLE 3:** Calculated intrinsic clearance (CL_int_) of RMB005, −059 and 060 using t_1/2_ in HLM and MLM and estimated *in vivo* hepatic clearance (CL_H_).

Compound	Degradation half-life (min)		*In vitro* CL_int_ (mL/min/kg)		Estimated *in vivo* CL_H_ (mL/min/kg)	
	MLM	HLM	MLM	HLM	MLM	HLM
RMB005	72.9	>150	104	4	81.2	12.6
RM059	16.6	10.6	457	410	75.2*	19.7*
RMB060	14.5	13.1	541	183	77.2*	18.6*

*Protein binding data not included.

MLM, mouse liver microsomes; HLM, human liver microsomes.

### 3.2 Pharmacokinetic assay performance

Quantification accuracy and precision were measured for the calibration range of the standard curve and the quality controls samples. Overall, the analytical methods were well suited for the PK analyses with standard and QC samples achieving an accuracy (%Nom) between 81.3 and 114.8% for all samples, with precision (%CV) below 15.5%, indicating good reproducibility. Curves were best fitted with quadratic regressions as the peak are ratio (drug/internal standard) against concentrations with 1/concentration with a weighting factor 1/x. Correlation coefficients for all curves were ≥0.99.

### 3.3 *In vivo* pharmacokinetic profiling

**FIGURE 4:**
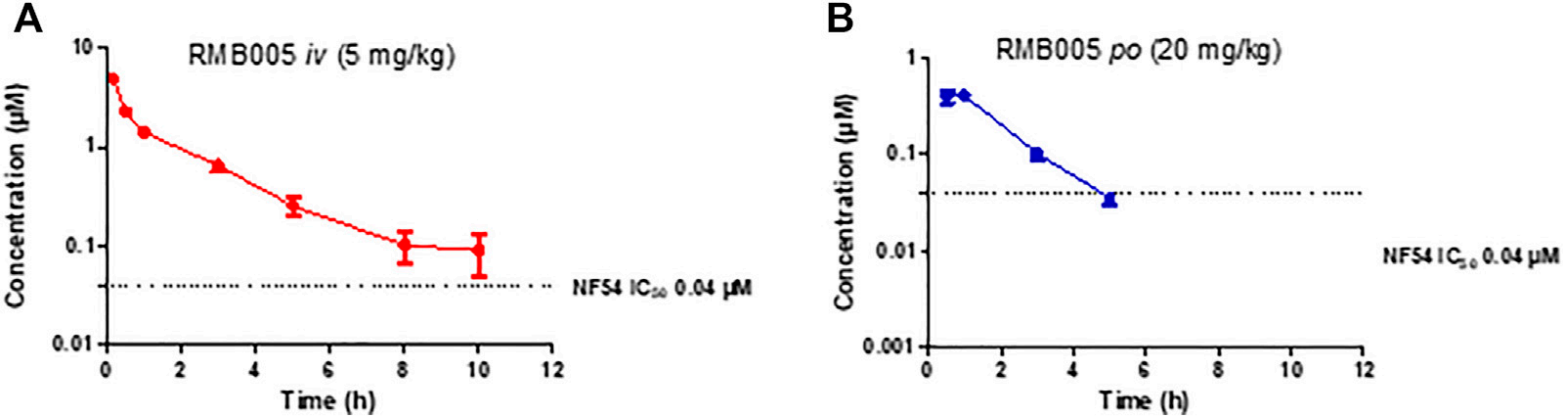
Mean ± SEM concentration-time profiles of RMB005 following **(A)** *iv* and **(B)** *po* administration. The dotted line represents IC_50_ activity of RMB005 against *Pf* NF54.

**FIGURE 5:**
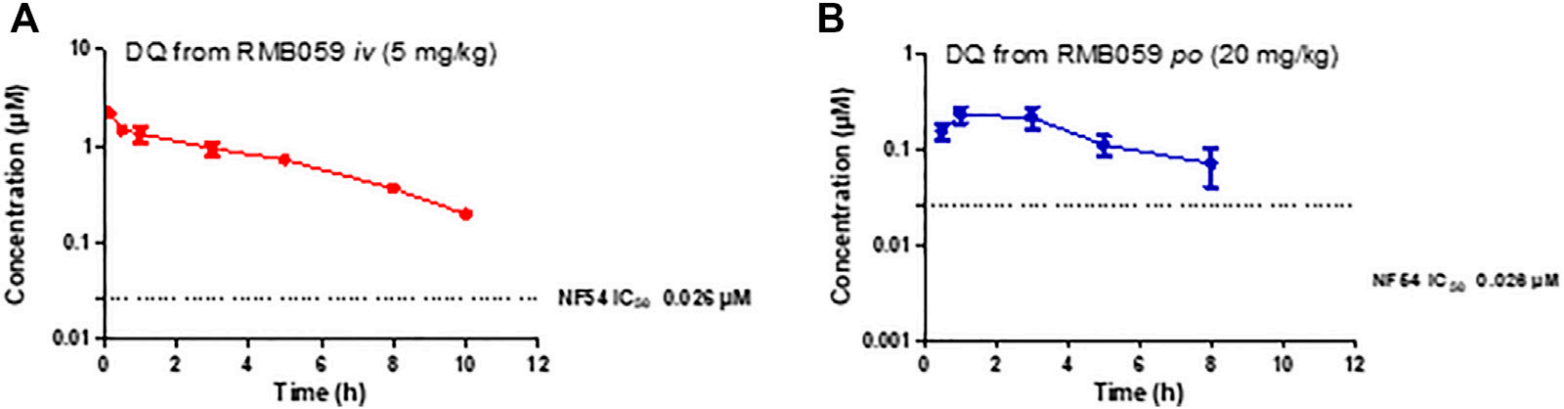
Mean ± SEM concentration-time profiles of DQ from RMB059 following **(A)** *iv* and **(B)** *po* administration. The dotted line represents IC_50_ activity of DQ against *Pf* NF54.

**FIGURE 6:**
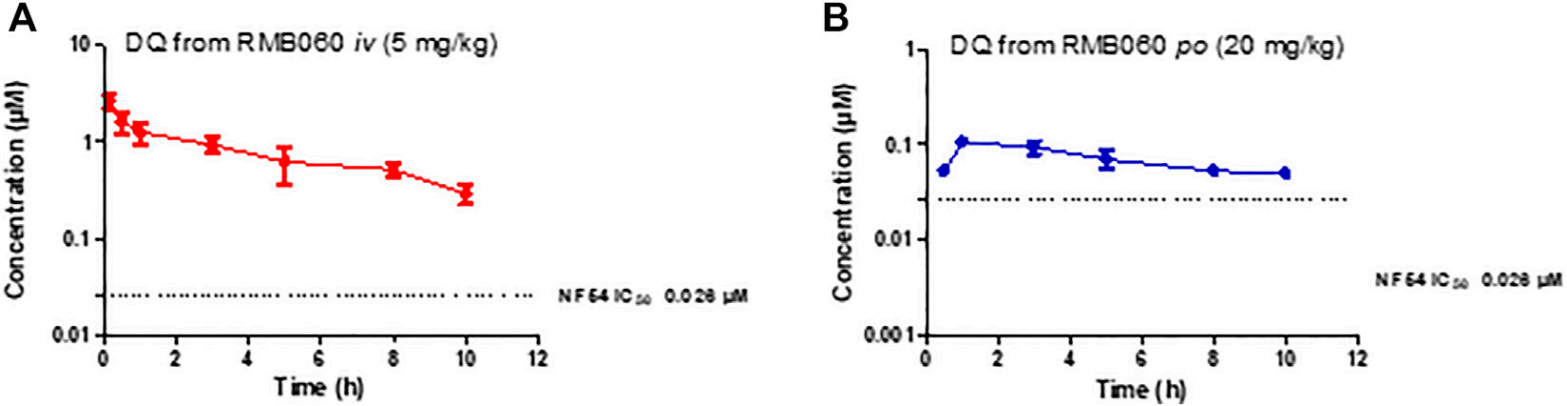
Mean ± SEM concentration-time profiles of DQ from RMB060 following **(A)** *iv* and **(B)** *po* administration. The dotted line represents IC_50_ activity of DQ against *Pf* NF54.

The whole blood concentration profiles of RMB005, −059 and −060 obtained from *iv* (*n* = 5) and *po* (*n* = 5) dosing groups in the murine model are presented in Figures 4–6. Data from plasma stability and method validation assays above revealed that the quinoline *O*-carbamates RMB059 and −060 undergo significant conversion into DQ in biological matrices. To overcome this problem, DQ was also analysed and quantified from the murine samples. Non-compartmental analysis was used to determine the parameters listed in Table 4. The IC_50_ of the NF54 parasite strain is indicated by the dashed line; in Figure 4 this is for RMB005, that does not decompose, in Figures 5, 6, the dotted line represents the IC_50_ for DQ. Because of decomposition of RMB059 and RMB060 to DQ on exposure to plasma or blood, non-compartmental analysis of DQ was also carried out (Table 4).

**TABLE 4:** Summary of *in vivo* pharmacokinetic (PK) parameters after intravenous 4) and oral (*po*) administration in mice.

Compound	t_1/2_ (h)	T_max_ (h)	C_max_ (µM)	Vd (L/kg)	CL (ml/min/kg)	AUC_0-∞_ (min µmol/L)	BA (%)
Intravenous							
RMB005	2.62 ± 0.61	-	-	6.59 ± 1.83	28.50 ± 2.17	-	-
DQ from RMB059	4.79 ± 1.66	-	-	8.00 ± 2.96	19.40 ± 3.14	-	-
DQ from RMB060	4.66 ± 1.16	-	-	8.88 ± 2.87	21.50 ± 3.38	-	-
Oral							
RMB005	-	0.70 ± 0.12	0.49 ± 0.02	-	-	59 ± 2	4 ± 0.3
DQ from RMB059	-	1.67 ± 0.67	0.23 ± 0.05	-	-	101 ± 44	nd
DQ from RMB060	-	1.67 ± 0.67	0.11 ± 0.01	-	-	63 ± 9	nd

AUC, area under the concentration-time curve; CL, clearance; C_max_, maximum plasma concentration; T_max_, time to reach C_max_; Vd, volume of distribution; *iv* and *po* administered doses of all compounds were 5 mg/kg and 20 mg/kg, respectively.

## 4 Discussion

The quinolone derivatives RMB005 and RMB059 were poorly soluble across all pH levels tested (<5 μM), and the results were comparable to the parent compound DQ (0.14 μM) (Bampidis et al., 2019). The ester carbamate RMB060 was unstable in solution and reliable evaluation was not possible. RMB059 and RMB060 were considered to have ideal lipophilicity (LogD_7.4_ 2.9) which is considered to be a LogD value between 1 and 3. RMB005 had lipophilicity slightly above the ideal range (LogD_7.4_ 3.2). However, all three compounds had improved lipophilicity over DQ (LogD of 7.8) (Burger et al., 2018). Passive permeability was classified as moderate (P_app_ between −5.5 and −6.5) for all three compounds tested. This was potentially due to the poor solubility at pH 6.5, which could have limited the amount of compound available in solution to cross the artificial membrane.

While RMB005 had good plasma stability, RMB059 and −060 showed a high degree of lability during assay of the plasma stability (Figures 2, 3). It was found that these compounds were rapidly converted into DQ. This finding has significant implications for the integrity of the compound in the systemic circulation during the *in vivo* experiments. However, this may well be advantageous, as DQ is known to be poorly absorbed. Therefore, if RMB059 or −060 presented improved absorption, they could act as prodrugs for delivery of DQ. This effect may also improve the efficacy profile of the quinolones as there will be both parent and converted active metabolite, that is, DQ, in circulation to increase the extent and duration of efficacy.

The mean whole blood concentration of RMB005 reached a maximum of 0.49 µM at approximately 0.70 h (Table 4), following a single oral dose of 20 mg/kg. Circulating concentrations remained above the *in vitro* IC_50_ (0.04 µM, Figure 4) for approximately 3 h. The whole blood concentrations were, however, below the reliable limit of quantification after 5 hours and fell below that of the IC_50_. The *in vivo* profile of RMB005 correlates well with the predicted *in vitro* ADME parameters in terms of solubility and exposure. Although RMB005 was quickly absorbed, the extent of exposure indicated by the low calculated AUC (59 min µmol/L) was presumably limited by poor solubility (<5 µM). CL was classified as relatively low (28.50 mL/min/kg), and the t_1/2_ was relatively short (2.62 h). The mean AUC_0-∞_ was 377 min µmol/L and the BA was low (4%). The calculation of CL_int_ which corrected for the free fraction (f_u_ 0.08) produced a CL value much higher than the original parameter (356 mL/min/kg). As RMB005 is moderately to highly bound to plasma proteins, the inclusion of the unbound fraction allows for more appropriate interpretation of the compound being metabolised in the system (Smith et al., 2018) and is reflected by the short t_1/2_ of 2.62 h the BA of RMB005 (4%) was similar to that of DQ which has been reported to have a relative BA of 6% when dosed as a micro-suspension in mice (Wang et al., 2014). Unfortunately, the poor PK properties of this compound precludes further investigation.

The mean C_max_ of DQ after oral dosing of RMB059 and −060 was 0.23 and 0.11 µM, respectively, and circulating levels of DQ were low overall as indicated by the low AUC values (Table 4). Systemic concentrations of DQ from both compounds remained above the IC_50_ (0.026 µM, Figures 5, 6) for at least 8 h. Concentrations fell below the LLOQ for DQ-RMB059 oral samples at 8 h, while that of the i.v. and both DQ-RMB060 groups fell below LLOQ at 10 h. CL of DQ from RMB059 and -060 was relatively slow (19.40 and 21.50 mL/min/kg, respectively). With reference to the rationale for investigating the derivatives RMB059 and RMB060, it is concluded that these compounds did not offer improved solubility, systemic exposure, or metabolic stability when compared to RMB005, even though enhancing BA was expected from the incorporation of carbamates (Igarashi et al., 2007). RMB059 and RMB060 were converted to DQ at a significant rate. Additionally, the exceptionally poor solubility of DQ in water (Wang et al., 2014) further limited the extent of DQ absorption. From this, it can be concluded that the ethyl ester carbamate quinolone DQ derivative series need optimization in terms of both in terms of solubility, and in enhancing the stability of certain metabolic ‘soft-spots’ such as the esters, amides, and carbamates which are more susceptible to hydrolysis (Di et al., 2005).

## 5 Conclusion

The RMB compounds derived from DQ were evaluated for their potential as longer acting third partner compounds to the amino-artemisinin-redox drug combination due to their nanomolar antimalarial activity against *P. falciparum*, ranging between 1.5 and 40.4 nm. The amide derivative of DQ, RMB005 was expected to show improved physicochemical properties to DQ, but it turned out to have poor solubility and permeability, resulting in very low BA (4%). The carbamate derivatives RMB059 and RMB060 were expected to show improved physicochemical properties over RMB005. However, these compounds turned out to be very unstable under physiological conditions and were rapidly converted to DQ under the assay conditions. Thus, future work will focus on preparing more stable O-quinoline carbamate and related derivatives from DQ.

## Data Availability

The raw data supporting the conclusions of this article will be made available by the authors, without undue reservation.
